# The Role of Chaperone-Mediated Autophagy in Bortezomib Resistant Multiple Myeloma

**DOI:** 10.3390/cells10123464

**Published:** 2021-12-08

**Authors:** Nicholas Nikesitch, Patricia Rebeiro, Lye Lin Ho, Srinivasa Pothula, Xin Maggie Wang, Tiffany Khong, Hazel Quek, Andrew Spencer, Cheok Soon Lee, Tara L. Roberts, Silvia C. W. Ling

**Affiliations:** 1 School of Medicine, Western Sydney University, Campbelltown, NSW 2560, Australia; nnikesitch@prostatecentre.com (N.N.); Tara.Roberts@westernsydney.edu.au (T.L.R.); soon.lee@westernsydney.edu.au (C.S.L.); 2 Ingham Institute of Applied Medical Research, Liverpool, NSW 2170, Australia; Patricia.Rebeiro@health.nsw.gov.au (P.R.); Srinivasa.Pothula@inghaminstitute.org.au (S.P.); 3 Vancouver Prostate Centre, Department of Urologic Sciences, University of British Columbia, Vancouver, BC V5Z 1M9, Canada; 4 Department of Haematology, Liverpool Hospital, Liverpool, NSW 2170, Australia; LyeLin.Ho@health.nsw.gov.au; 5 School of Medicine, University of New South Wales, Kensington, NSW 2033, Australia; 6 NSW Health Pathology, Liverpool Hospital, Pathology Building, Liverpool, NSW 2170, Australia; 7 Westmead Institute for Medical Research, The University of Sydney, Westmead, NSW 2145, Australia; xin.wang@sydney.edu.au; 8 Malignant Haematology & Stem Cell Transplantation Service, Alfred Hospital, Melbourne, VIC 3004, Australia; tiffany.khong@monash.edu (T.K.); andrew.spencer@monash.edu (A.S.); 9 Australian Centre for Blood Diseases, Monash University, Melbourne, VIC 3004, Australia; 10 QIMR Berghofer Medical Research Institute, Herston, QLD 4006, Australia; Hazel.Quek@qimrberghofer.edu.au; 11 Department of Anatomical Pathology, Liverpool Hospital, Sydney, NSW 2170, Australia; 12 Faculty of Medicine, University of Queensland Centre for Clinical Research, Herston, QLD 4006, Australia

**Keywords:** multiple myeloma, resistance, bortezomib, chaperone mediated autophagy, LAMP2A, ER stress and autophagy

## Abstract

**Background:** Multiple myeloma (MM) remains incurable despite high-dose chemotherapy, autologous stem cell transplants and novel agents. Even with the improved survival of MM patients treated with novel agents, including bortezomib (Bz), the therapeutic options in relapsed/refractory MM remain limited. The majority of MM patients eventually develop resistance to Bz, although the mechanisms of the resistance are poorly understood. **Methods:** Lysosomal associated membrane protein 2A (LAMP2A) mRNA and protein expression levels were assessed in ex vivo patient samples and a Bz-resistant MM cell line model by in real-rime PCR, western blotting and immunohistochemistry. In vitro modelling of chaperone-mediated autophagy (CMA) activity in response to ER stress were assessed by western blotting and confocal microscopy. The effects of CMA inhibition on MM cell viability and Bz sensitivity in MM cells were assessed by Annexin V/7AAD apoptosis assays using flow cytometry. **Results:** In this study, there is evidence that CMA, a chaperone-mediated protein degradation pathway, is upregulated in Bz-resistant MM and the inhibition of CMA sensitises resistant cells to Bz. The protein levels of LAMP2A, the rate-limiting factor of the CMA pathway, are significantly increased in MM patients resistant to Bz and within our Bz-resistant cell line model. Bz-resistant cell lines also possessed higher basal CMA activity than the Bz-sensitive parent cell line. In MM cell lines, CMA activity was upregulated in response to ER stress induced by Bz. The inhibition of CMA sensitises Bz-resistant cells to Bz and the combination of CMA inhibition and Bz in vitro had a more cytotoxic effect on myeloma cells than Bz alone. **Conclusion:** In summary, the upregulation of CMA is a potential mechanism of resistance to Bz and a novel target to overcome Bz-resistant MM.

## 1. Introduction

Multiple myeloma (MM) remains an incurable malignancy despite high-dose chemotherapy, autologous stem cell transplants and novel agents. MM is a genetically heterogeneous disease with increasing genetic complexity as the disease progresses to a more aggressive stage [1]. Myeloma cells are highly dependent upon the unfolded protein response (UPR) to modulate ER stress levels and restore cellular proteostasis caused by excessive paraprotein production [2]. The UPR modulates ER stress via a number of mechanisms, but myeloma cells are highly dependent upon the proteasomal degradation of paraprotein to reduce proteotoxic ER stress [3]. This has made the treatment of MM with proteasome inhibitors (PIs) such as bortezomib (Bz) one of the most effective ways of treating the disease, significantly improving the survival of MM patients to 4–6 years [4]. However, relapse refractory MM still remains the biggest hurdle in long-term survival, as the majority of MM patients eventually develop a resistance to Bz [4]. The causes of Bz resistance remain poorly understood; however, sensitivity to Bz is well reported to be mediated by the dependence myeloma cells have on the UPR, with reduced X-box binding protein 1 (XBP1) and activating transcription factor 6 (ATF6) expression mirroring Bz resistance [5,6]. The reduced UPR function suggests an alternative stress mechanism that is responsible for compensating the UPR and alleviating ER stress in Bz resistance.

Chaperone-mediated autophagy (CMA) is a highly specific pathway involved in the degradation of soluble cytosolic proteins and could potentially be responsible for alleviating ER stress and conferring Bz resistance. The activity of the pathway is dependent upon the expression of the lysosome-associated membrane protein 2A (LAMP2A), [7,8]. Upregulated in a number of tumours [9,10,11], CMA is also reported to promote tumour cell proliferation and metastasis in lung and breast cancer, but also plays a pivotal role in doxorubicin resistance in breast cancer [9,11,12]. Breast cancer cells deficient of LAMP2A also possess increased sensitivity to doxorubicin [11]. However, CMA is yet to be studied in MM and/or Bz resistance. It is already well established that CMA is upregulated under oxidative, metabolic, proteotoxic and genotoxic stress [13,14,15], making it an important stress mechanism for cell survival.

This study aims to determine the importance and role of CMA in Bz resistance in MM and whether inhibition of CMA sensitises resistant cells to Bz.

## 2. Methods

### 2.1. Reagents

Anti-LAMP2A (ab18528), β-actin (ab8227), rabbit IgG H&L (ab16284), rabbit IgG isotype control (ab27478) and Alexa 555 anti-rabbit IgG (ab150074) were purchased from Abcam (Cambridge, UK). Anti-CD38-APC (340439) was purchased from BD Biosciences (Franklin Lakes, NJ, USA). Anti-CD138-PE (A40316) was purchased from Beckman Coulter (Lane Cove West, NSW, Australia). Anti-CD138 (MA5-12400) and Alexa 488 anti-mouse IgG (A-11029) were purchased from Life Technologies (Victoria, Australia). Anti-HSC70 was purchased from Novus (Centennial, CO, USA). All other reagents were purchased from Sigma-Aldrich (Sigma-Aldrich, St. Louis, MO, USA), unless otherwise stated.

### 2.2. Cell Culture

Myeloma cell lines, KMS-11, U266, RPMI 8226 and OPM-2 were provided by Professor Andrew Spencer. Cells were cultured using RPMI-1640 medium (Sigma-Aldrich, St. Louis, MO, USA) supplemented with 10% foetal bovine serum (FBS) (Gibco, Life Technologies, Victoria, Australia), 5% L-glutamine (Sigma) and 1% penicillin streptomycin (PenStrep) (Sigma) and at 37 °C in a 5% CO_2_ atmosphere. KMS11 bortezomib-resistant cells were previously developed by Dr. Silvia Ling and were cultured in the presence of bortezomib (Selleckchem PS-341) (Houston, TX, USA) as previously described [5].

### 2.3. Patient Samples

Twenty-nine bone marrow aspirate samples were collected from MM patients prior to the treatment with Bz from Liverpool Hospital, NSW Australia, with written and informed consent, and approved by the South Western Sydney Local Health District Human Research Ethics Committee. The patient characteristics are provided in the supplementary data (Supplementary Table S1). Bone marrow mononuclear cells were isolated and cell sorted as previously described [6], with patient clinical responses to Bz determined according to the International Myeloma Working Group (IMWG) uniform response criteria [16]. A total of 66 MM patient bone marrow trephines were obtained from the Australian Centre for Blood Diseases, Monash University (provided by Professor Andrew Spencer), with written and informed consent. The protocol was approved by the South Western Sydney Local Health District Human Research Ethics Committee and the Monash University Human Research Ethics Committee. The cohort consisted of complete response/very good partial response (CR/VGPR) (n = 20), partial response (PR) (n = 22), minimal response (MR) (n = 11), stable disease (SD) (n = 6) and progressive disease (PD) (n = 7). Trephine samples were collected prior to patients undergoing any proteasome inhibitor treatment. Patient clinical responses to proteasome inhibitors were determined according to the IMWG uniform response criteria.

### 2.4. Gene Expression Profiling of LAMP2A by Real-Time PCR

RNA extraction and cDNA synthesis were performed as previously described [6]. LAMP2A gene expression was quantitated by real-time PCR on the Rotor-Gene Q (Qiagen, Hilden, Germany) using gene specific primers developed by Cacciottolo et al. (2013). LAMP2A was normalised against the GAPDH housekeeping control for each sample using the GAPDH QuantiTect Primer Assay (Qiagen-QT01192646), and the relative gene expression calculated using the ΔΔCt Method [17]. Reactions were amplified using the following thermal cycling profile: 95 °C (5 min), followed by 40 cycles of 95 °C (10 sec), 61 °C (30 s). Melt curve analysis was performed on all samples. Each run included no template controls.

### 2.5. Western Blotting

Cells were lysed with RIPA buffer (Sigma), containing protease inhibitor cocktail (Sigma) and protein concentrations determined using a Pierce Coomassie Protein Assay Kit (Thermo Scientific), following the manufacturer’s instructions. Samples were subjected to SDS-PAGE using 4–20% Mini-PROTEAN TGX Precast Gels (BIORAD; Hercules, California, USA) and transferred to Immobilon-P 0.2 μm PVDF membrane (EMD Millipore; Burlington, Massachusetts, USA). Membranes were blocked in 5% (w/v) skim milk in tris-buffered saline (TBS) and 0.1% Tween20 (TBST), and then left to incubate with primary antibodies overnight at 4 °C. Membranes were washed with 0.1% TBST, followed by secondary blotting for 60 min at 4 °C. Membranes were subsequently washed with 0.1% TBST and treated with Clarity Western ECL Substrate solution (BIORAD) for 5 min prior to imaging using a VersaDoc Imaging System (BIORAD) or Licor Odyssey Fc Imaging System (Li-COR) where specified. Protein expression was quantified using ImageJ 1.46r software.

### 2.6. Immunofluorescence

A serum-starved control (18–24 h) was cultured for each experiment to maximally activate CMA [18]. Harvested cells were resuspended in PBS, with 10,000–20,000 cells cytospun onto glass coverslips (Thermo Scientific; Waltham, Massachusetts, USA) using a Cytospin 4 (Thermo Scientific) at 1500 rpm for 3 min. Cells were then fixed with 50% methanol for 2 min, then 100% methanol (−20 °C) overnight. Upon staining, samples were gradually rehydrated with phosphate buffered saline (PBS), and then rinsed with PBS (x3). Samples were blocked for 30 min (2% FCS, 0.3 M glycine, 1% BSA, and 0.01% Triton X-100 in PBS), rinsed with PBS and then incubated with primary antibodies for 90 min. Secondary antibody only negative controls were included for each experiment. Coverslips were washed with PBS with 0.2% Tween 20 (PBST) and incubated with secondary antibodies for 60 min. Coverslips were washed with 0.2% PBST for 5 min and mounted using Prolong Gold Antifade Mountant with DAPI (Life Technologies). Images were captured by confocal microscopy using a Leica SP5 confocal microscope (Leica Biosystems; Wetzlar, Germany). Quantification of LAMP2A/HSC70 colocalised puncta (CMA active lysosomes) was performed on Z-stack images using ImageJ software (NIH). Colocalisation analysis and the counting of the number of colocalised puncta were performed using the ImageJ JACoP Plugin [19]. Pearson coefficients (r) for the colocalisation of LAMP2A and HSC70 on lysosomes were generated using the ImageJ JACoP Plugin.

### 2.7. IHC of Bone Marrow Trephines

Bone marrow trephines were cut in 4 μm sections and mounted on Superfrost Plus slides (Thermo Scientific). All immunohistochemical staining was performed using the EnVision FLEX High pH kit (Dako; Santa Clara, CA, USA), following the manufacturer’s protocol with minor modification, unless stated otherwise. Slides were scanned at x40 magnification and converted into digital images using the Aperio AT Turbo Pathology Digital Scanner (Leica Biosystems). The staining intensity and extent of staining of LAMP2A was scored in CD138 positive stained cells of superimposed images for each bone marrow trephine. Scoring was performed blinded by 3 independent scorers, 2 of which were haematopathologists. Staining intensity was scored on a 1–3 scale, 1 = low intensity, 2 = medium intensity and 3 = high staining intensity. The extent of staining was scored as follows: 1 = 0–25%, 2 = 26–50%, 3 = 51–75% and 4 = 76–100%. Both scores were multiplied to give an overall score for each section.

### 2.8. Apoptosis Assays

Apoptosis was assessed using a PE Annexin V Apoptosis Detection kit (BioLegend; Western Australia, Perth). Cell suspensions were collected and adherent cells were then harvested using 2 mM EDTA. Harvested cells were pelleted, then subsequently washed with PBS and pelleted. Cell pellets were suspended in Annexin V binding buffer and stained with 7-AAD and PE Annexin V as recommended by the manufacturer. Samples were analysed by flow cytometry using a BD FACSCanto II (BD Biosciences). For each experiment, the following controls were included in order to identify each cell population, which included an unstained control (live population), 7AAD control (necrotic population) and Annexin V control (apoptotic population). Cells used for the 7AAD control were combined with 0.1% saponin in order to permeablise the cells. Data was analysed using the BD FACSDiva™ software.

### 2.9. Statistical Analysis

All graphs were generated using GraphPad Prism (GraphPad Software, CA, USA) and statistical analysis was performed by unpaired *t*-test with Welch’s correction, student *t*-test, Mann–Whitney *U* test or one-way ANOVA.

## 3. Results

### 3.1. CMA Is Upregulated in Bortezomib-Resistant Multiple Myeloma

**Figure 1:**
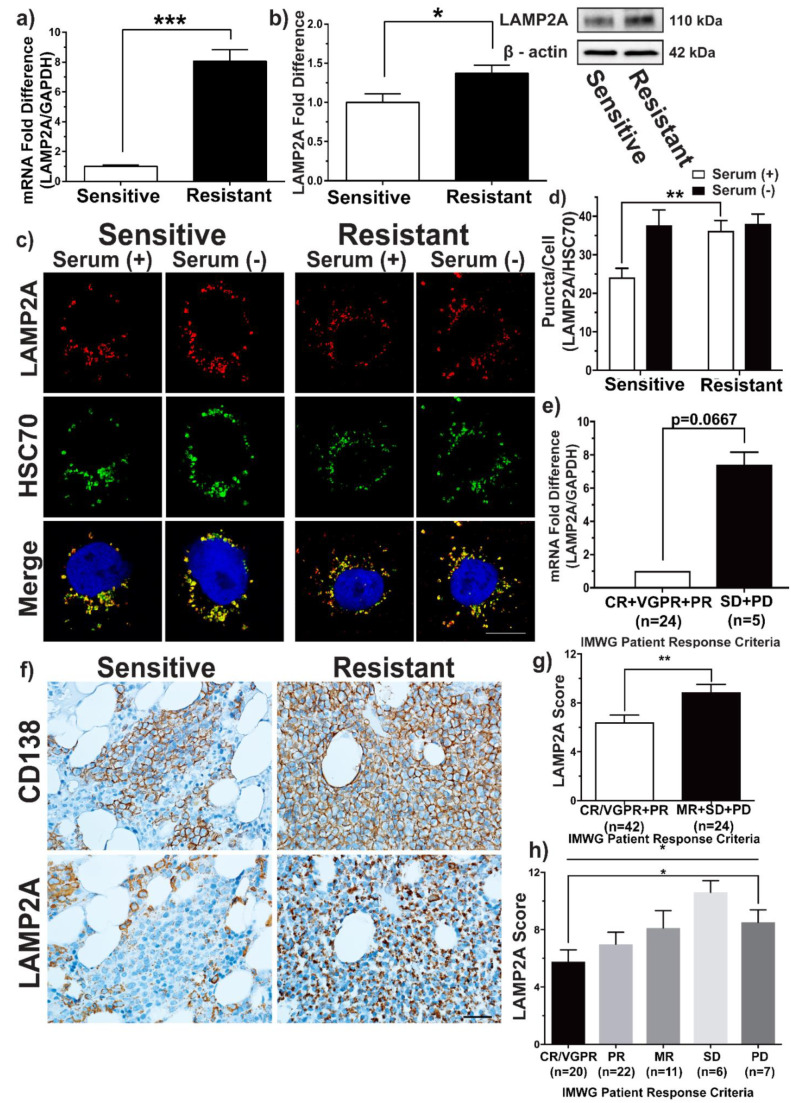
Analysis of CMA in bortezomib-sensitive and resistant multiple myeloma. Relative fold difference in LAMP2A expression between KMS11-sensitive (white) and resistant cells (black). (**a**) mRNA expression. Data shown as mean ± S.E.M (n = 6; *** *p* = 0.0002, *t*-test). (**b**) Protein expression. Data shown as mean ± S.E.M (n = 6; * *p* = 0.0329, *t*-test). (**c**) KMS11 bortezomib-sensitive (left panel) and resistant cells (right panel) cultured in the presence (Serum +) or absence (Serum -) of serum for 24 h. Cells were fixed and probed for LAMP2A (red) and HSC70 (green). Serum deprived controls were included for each cell type to maximally active CMA. Merged LAMP2A/HSC70 is shown in the bottom row (yellow). Scale bar is 10 μm. (**d**) Quantification of the mean number of CMA active (LAMP2A/HSC70) puncta per cell ± S.E.M (** *p* = 0.0015; *t* test); Pearson coefficient means (from left to right on graph): r = 0.8; r = 0.77; r = 0.75; r = 0.7. For each experiment, ≥10 cells per condition were analysed (n = 3). (**e**) LAMP2A mRNA expression in bortezomib resistant patients (black) (n = 5) relative to bortezomib-sensitive patients (white) (n = 24). Patients were grouped according to the IMWG Patient Response Criteria. Data presented as mean values ± SD. Statistical analysis was performed on sensitive patients vs. resistant patients (*p* = 0.0667, Mann–Whitney *U* test). (**f**) Immunohistochemistry staining of CD138 (brown stain) (top row) and LAMP2A (brown stain) (bottom row) in bortezomib-sensitive (left panel) and resistant (right panel) multiple myeloma patient bone marrow trephines. Objective x40. Scale bar 50 μm. (**g**) LAMP2A protein expression in bortezomib-sensitive patients (CR/VGPR + PR; n = 42) (white) and bortezomib-resistant patients (MR + SD + PD; n = 24) (black). Data is shown as mean values ± S.E.M. Statistical analysis was performed on sensitive patients vs. resistant patients (** *p* = 0.0071, *t* test). (**h**) Individual means of LAMP2A expression for each patient group from (**g**). Data is shown as mean values ± S.E.M. Statistical analysis was performed on CR/VGPR (n = 20) vs. PR (n = 22) vs. MR (n = 11) vs. SD (n = 6) vs. PD (n = 7) using one-way ANOVA (* *p* = 0.0454 (top), * *p* = 0.0319 (bottom)).

Under increasing Bz resistance, the expression levels of important regulators of the UPR are known to decrease in expression in MM [5,20,21]. Therefore, an alternative stress mechanism is likely responsible for alleviating ER stress in myeloma cells that possess reduced UPR activity, and hence contributing to Bz resistance. We hypothesised that CMA is responsible in alleviating ER stress in myeloma cells that are resistant to Bz. Considering that LAMP2A is the rate-limiting factor of CMA activity, we first quantified LAMP2A mRNA and protein expression in a Bz-sensitive and Bz-resistant MM cell line model. We identified that LAMP2A mRNA expression was significantly higher in resistant cells compared to the parent sensitive cells (Figure 1a; *p* = 0.0002). We went on to further examine the protein expression levels within KMS11-sensitive and resistant cells by western blot analysis by quantifying the heavily glycosylated 110–120 kDa LAMP2A isoform, which resides in the membrane of the lysosome and mediates CMA activity [22]. KMS11-resistant cells displayed an increase in LAMP2A protein expression relative to sensitive cells (Figure 1b; *p* = 0.0329). The increase in both LAMP2A protein and mRNA expression in the resistant cells indicated that CMA activity was upregulated in Bz resistance.

We were able to further confirm our early data by assessing basal CMA activity in KMS11-sensitive and resistant cells by confocal microscopy. CMA competent and active lysosomes are characterised by possessing both HSC70 and LAMP2A [18], and therefore used to quantify the number of CMA active lysosomes represented by colocalised LAMP2A/HSC70 (heat shock conjugate 70) puncta. Basal CMA activity in sensitive and resistant cells was determined by comparing basal CMA levels to the corresponding serum-starved controls. Serum starving cells for 24 h allowed CMA to be fully activated, enabling the total number of possible active lysosomes to be determined.

Under basal conditions, the resistant cells displayed a substantially higher number of CMA active lysosomes (HSC70/LAMP2A localized puncta) compared to the sensitive cells by 33.35% (Figure 1c,d; *p* = 0.0015). Basal CMA activity of resistant cells was the highest of the two cell lines, measuring at 95.29% of the maximum active capacity. CMA levels within the sensitive cells were considerably lower, measuring 64.05% of the maximum active capacity of CMA. Under serum-starved conditions, in which CMA plateaus reaching maximum activity, both cell lines were observed to have an almost identical number of HSC70/LAMP2A localised puncta as expected.

To characterise these findings translationally, we next assessed LAMP2A mRNA expression in bone marrow aspirates of 29 patients prior to undergoing Bz treatment. The mRNA expression of LAMP2A was correlated with patient’s clinical response to Bz after cycle 2 according to the IMWG uniform response criteria [16]. Resistant patients that were classified as having either stable disease (SD) or progressive disease (PD) were assigned to the resistant patient cohort. The sensitive patient cohort consisted of patients who had very good partial response (VGPR) or partial response (PR). There was a trend that the median LAMP2A mRNA expression of the resistant group (n = 5) was higher than the sensitive group (n = 24) (Figure 1e; *p* = 0.0667, Mann–Whitney *U* test), though not statistically significant.

We further examined LAMP2A protein expression in the bone marrow trephines of an independent cohort of 66 MM patients by immunohistochemistry. For each bone marrow trephine, slides were stained with LAMP2A, with an additional slide from adjacent section stained for CD138 to accurately locate and identify myeloma cell populations within patient bone marrow trephines for LAMP2A scoring [23]. Patients resistant to Bz (MR + SD + PD; n = 24) expressed higher LAMP2A protein levels compared to patients sensitive to Bz (CR/VGPR + PR; n = 42) (Figure 1f,g; *p* = 0.0071). Relative to the sensitive patients, myeloma cells in resistant patients displayed a 1.38-fold increase in LAMP2A protein expression. Between individual patient groups CR/VGPR (n = 20) vs. PR (n =22) vs. MR (n = 11) vs. SD (n = 6) vs. PD (n = 7), LAMP2A appeared to increase in expression as Bz resistance increased (Figure 1h; *p* = 0.0454). However, only CR/VGPR vs. PD were found to be significantly different (*p* = 0.0319).

### 3.2. CMA Increases under ER Induced Stress

**Figure 2:**
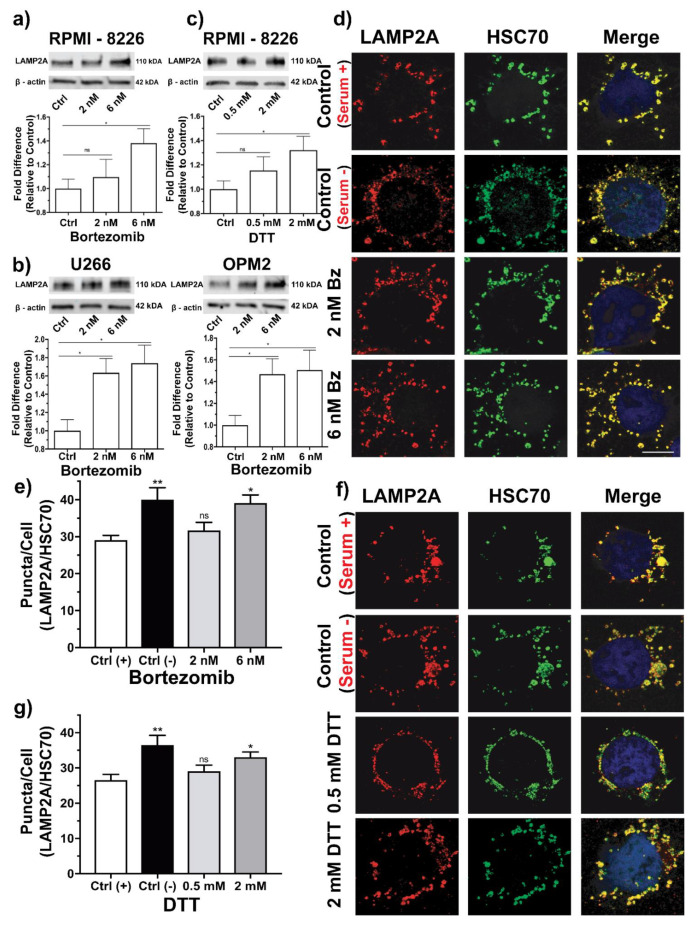
Upregulation of CMA in response to ER-induced stress. Western blot densitometry analysis of LAMP2A protein expression in (**a**) RPMI 8226 cells treated with bortezomib for 24 h. Data shown as mean ± S.E.M (n = 8; *p* = ns, * *p* = 0.0218, *t*-test). (**b**) U266 and OPM2 cells treated with bortezomib for 24 h. Data shown as mean ± S.E.M (U266; n = 4; * *p* = 0.0183, *p* = 0.023, *t* test) (OPM2; n = 5; * *p* = 0.028, *p* = 0.05, *t*-test). (**c**) RPMI 8226 cells treated with DTT for 24 h. Data shown as mean ± S.E.M (n = 6; *p* = ns, * *p* = 0.0397, *t*-test). (**d**) Confocal microscopy of RPMI 8226 cells treated with bortezomib for 24 h. Each experiment included an untreated control (with serum) and a serum deprived control (without serum) to maximally activate CMA. Cells were fixed and probed for LAMP2A (red) and HSC70 (green) for confocal imaging. Merged LAMP2A/HSC70 is shown in the last row (yellow). For each experiment, up to ≥10 cells per condition were analysed in three independent experiments. Objective x63. Scale bar is 10 μm. (**e**) Quantification of the mean number of LAMP2A/HSC70 colocalised puncta per cell ± S.E.M from Figure 2d. Data is presented as mean number of LAMP2A/HSC70 colocalised puncta per cell ± S.E.M (** *p* = 0.0051; * *p* = 0.0133; *p* = ns; one-way ANOVA); Pearson coefficient means (from left to right on graph): r = 0.89; r = 0.88; r = 0.89; r = 0.90. (**f**) Confocal microscopy of RPMI 8226 cells treated with DTT for 24 h. The same parameters used as in Figure 2d. (**g**) Data from Figure 2f presented as mean number of LAMP2A/HSC70 colocalised puncta per cell ± S.E.M (** *p* = 0.0015; * *p* = 0.0417; *p* = ns; one-way ANOVA); Pearson coefficient means (from left to right on graph): r = 0.88; r = 0.87; r = 0.83; r = 0.88.

We next aimed to determine if CMA was upregulated under ER-induced stress and functioning as a compensatory mechanism to alleviate ER stress. RPMI 8226, U226 and OPM2 MM cell lines were cultured and treated with two sublethal concentrations of Bz (2 nM and 6 nM) for 24 h, prior to LAMP2A protein expression analysis by western blotting. We observed a 38% increase in LAMP2A expression in RPMI 8226 cells subjected to 6 nM Bz compared to the untreated control (Figure 2a; *p* = 0.0196), while little difference was detected in cells treated with 2 nM Bz (*p* = ns). The U266 cell line (Figure 2b) appeared to have a greater change in LAMP2A protein expression in response to Bz, with increases of 63% (*p* = 0.0183) and 75% (*p* = 0.023) at 2 nM and 6 nM, respectively, relative to the untreated control. Similar results were also observed at both concentrations in OPM2 cells (Figure 2b; *p* = 0.028 and *p* = 0.05). We went on further to show that increases in LAMP2A expression were a result of increased ER stress and not a result of proteasome inhibition. We therefore used dithiothreitol (DTT) to reduce the disulphide bonds within proteins causing the denaturing of proteins which resulted in ER stress [24]. However, due to the toxicity of DTT, cells were only treated for 5 h. Nevertheless, LAMP2A protein expression in response ER stress induced by DTT were similar to those seen in RPMI 8226 cells subjected to ER stress induced by Bz (Figure 2c). Cells treated with 2 mM DTT displayed the greatest increase in LAMP2A protein expression of up to 32% (*p* = 0.0397).

We also identified an increase in the number of CMA-active lysosomes in RPMI 8226 cells subjected to ER-induced stress by either Bz or DTT using confocal microscopy. Relative to the untreated control (with serum), the number of colocalised HSC70/LAMP2A puncta were increased by 26% within cells treated with 6 nM Bz (Figure 2d,e; *p* = 0.0133). However, there was no significant difference in cells treated with 2 nM Bz compared to the untreated control (*p* = ns). Relative to serum-starved controls (maximum CMA activity), cells treated with 6 nM Bz almost reached maximum CMA activity, 2% less than the maximum CMA active capacity. Under basal conditions (with serum), basal CMA levels of the untreated controls was 73% of active capacity (*p* = 0.0051), slightly less than 2 nM treated cells (*p* = ns). ER-induced stress using DTT also increased the number of CMA active lysosomes (Figure 2f,g). The degree of CMA activity within cells treated with DTT was similar to the responses seen in Bz-treated cells. CMA levels increased by 9% in cells treated with 0.5 mM DTT (*p* = ns), and almost 20% (*p* = 0.0417) in cells treated with 2 mM DTT, relative to the untreated control. Cells treated with 2 mM DTT reached 90.5% active capacity, while basal CMA levels of untreated cells and CMA levels of serum starved cells remained relatively consistent in Bz and DTT experiments.

In summary, CMA activity is upregulated in MM under ER stress induced by Bz or DTT and plays an important role as a stress mechanism in alleviating proteotoxicity. It also appears to serve as a compensatory stress mechanism during instances where ER stress levels exceed the UPRs functional threshold.

### 3.3. CMA Inhibition Enhances Bz Sensitivity in MM Cells

Increased CMA activity and LAMP2A expression in Bz-resistant myeloma cells and upregulation of CMA under ER stress demonstrates CMA’s importance in alleviating ER stress and maintaining proteostasis. CMA is negatively regulated by Akt and mTOR Complex 2 (mTORC2), which illicit an inhibitory effect on CMA activity by phosphorylating Glial Fibrillary Acidic Protein (GFAP). Dephosphorylation of Akt by PH domain and Leucine-rich repeat protein phosphatase (PHLPP1) counters the inhibitory effect that mTORC2 and Akt has on CMA activity [25], enabling GFAP to stabilize LAMP2A in the translocation complex [26]. We investigated the effects of CMA inhibition (CMAi) on Bz resistance through the inhibition of the phosphatase activity of PHLPP1 by a selective inhibitor molecule (NSC ID: 117079) (from here referred to as PHLPPi). This inhibitor has been previously shown to effectively inhibit CMA activity with almost complete inhibition at 30 μM in vitro [25].

Our previous experiments suggested that the IC50 of KMS11-sensitive cells were approximately 5 nM following 48 h of Bz treatment (data not shown). For the KMS11-sensitive cells, we treated the cells with two different concentrations of Bz, 3 nM and 6 nM. To determine the effects of CMA inhibition on Bz sensitivity, we tested the effect of PHLPPi (30 μM alone and in combination with both Bz concentrations). Cells were treated over 48 h, with cell viability analysed by flow cytometry at 24 and 48 h using Annexin V/7AAD staining. The same experimental procedure was carried out on KMS11 resistant cells but using higher Bz concentrations of 10 nM and 20 nM. The IC50 of KMS11-resistant cells was approximately 20 nM Bz after 48 h of treatment (data not shown). Bz concentrations of 10 nM and 20 nM were used, as it was hypothesised that CMA inhibition would reduce resistance to Bz.

**Figure 3:**
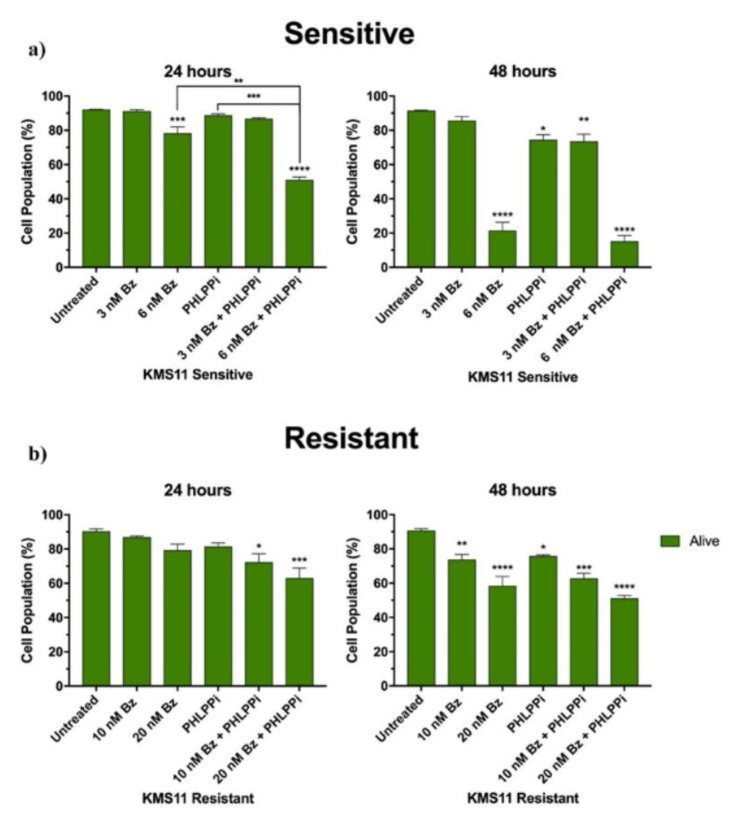
The effect of CMA inhibition on bortezomib-sensitive and resistant MM cells. Bortezomib-sensitive and resistant KMS11 cells were either treated with bortezomib, PHLPPi or treated in combination with the PHLPPi and bortezomib for 24 and 48 h. Viability of cells were analysed by flow cytometry at 24 and 48 h using PE-Annexin V (apoptotic cell marker) and 7-AAD (live cell exclusion stain) exclusion staining. For each experiment, a total of 10,000 cells were analysed per sample. Data is presented as mean population percentage ± S.E.M (n = 3). Statistical analysis was performed using a one-way ANOVA across all samples. (**a**) KMS11-sensitive cells (24 h: *** *p* = 0.0005; ** *p* = 0.0088; *** *p* = 0.0005; **** *p* = ≤ 0.00001) (48 h: **** *p* = ≤ 0.0001; * *p* = 0.0142; ** *p* = 0.01; **** *p* = ≤ 0.0001). (**b**) KMS11-resistant cells (24 h: * *p* = 0.0151; *** *p* = 0.0007) (48 h: ** *p* = 0.0069; **** *p* = ≤ 0.0001; * *p* = 0.0167; *** *p* = 0.0001; **** *p* = ≤ 0.0001).

For the KMS11 Bz-sensitive cells at 24 h (Figure 3a), there was a decrease in cell viability of <1% (*p* = ns) and 15% (*p* = 0.0005) in cells treated with 3 nM Bz and 6 nM Bz, respectively. The inhibition of CMA using the PHLPPi alone resulted in a small decrease in cell viability of 3.5% relative to the untreated control (*p* = ns). The combination of 3 nM Bz + PHLPPi led to a small reduction in cell viability of up to 6% (*p* = ns), while the combination of 6 nM Bz + PHLPPi resulted in a substantial reduction in cell viability of almost 45% (*p* = 0.0001). Therefore, 3 nM + PHLPPi led to a 2-fold increase in cytotoxicity compared with 3 nM of Bz, and 6 nM Bz + PHLPPi resulted in almost a 3-fold increase in cytotoxicity compared with 6 nM Bz. The combination of 6 nM Bz + PHLPPi had a substantially enhanced cytotoxic effect on the viability of the KMS11 cells compared with 6 nM Bz alone (*p* = 0.0088) and PHLPPi alone (*p* = 0.005). Interestingly, early apoptotic cell numbers were noticed to be higher in cells treated with Bz compared to cells treated with Bz + PHLPPi. The Bz + PHLPPi combination appeared to induce apoptosis more rapidly, which was indicated by higher late apoptotic cell numbers in Bz + PHLPPi treated samples (Supplementary Figure S1a). We identified a 34% (*p* = ns) and 50% (*p* = 0.0315) increase in late apoptotic cell numbers in 3 nM Bz + PHLPPi and 6 nM Bz + PHLPPi samples relative to the sensitive cells treated with 3 nM and 6 nM Bz. This suggests that PHLPPi in combination with Bz led to more rapid cytotoxicity.

Relative to the untreated controls, KMS11-sensitive cells at 48 hrs, there was almost a 6.5% (*p* = ns) and 77% (*p* = 0.0001) decrease in cell viability with 3 nM and 6 nM of Bz. Cytotoxicity was identified to be further enhanced by the combination of the PHLPPi + Bz in the sensitive cells, with 3 nM Bz + PHLPPi and 6 nM Bz + PHLPPi combinations reducing the viability by 20% (*p* = 0.01) and 83% (*p* = 0.0001) relative to the untreated control. There were substantially higher late apoptotic cell numbers within samples that were treated with Bz + PHLPPi than in samples that were treated with Bz alone.

Within KMS11 Bz resistant cells, PHLPPi sensitised the cells to Bz (Figure 3b). Bz treatment alone appeared to have little effect on the viability of the resistant cells after 24 h. Relative to the untreated control, the viability of the resistant cells was identified to decrease by 4% (*p* = ns) and 12% (*p* = ns) in response to 10 nM and 20 nM Bz. PHLPPi alone did not alter cell viability significantly, but treatment with 10 nM Bz + PHLPPi and 20 nM Bz + PHLPPi led to a significant reduction in cell viability by 20% (*p* = 0.0151) and 30% (*p* = 0.0007), respectively. Therefore, combining PHLPPi + 10 nM Bz, there was a 5-fold increase in cytotoxicity, and a 2.5-fold increase in cytotoxicity for PHLPPi + 20 nM. This suggests that the inhibition of CMA by PHLPPi sensitised the resistant cells to Bz. Apoptosis occurred more rapidly in resistant cells treated with Bz + PHLPPi compared to those treated with Bz or the PHLPPi alone (Supplementary Figure S1b). Resistant cells treated with Bz + PHLPPi possessed a larger number of late apoptotic cells than early apoptotic cells compared to samples treated with Bz alone, which were found to be mostly in the early stages of apoptosis.

After 48 h, 10 nM and 20 nM Bz reduced the viability of the resistant cells by 19% (*p* = 0.0069) and 36% (*p* = 0.0001) relative to the untreated control. The greatest cytotoxic effect on the resistant cells was seen in samples treated with 10 nM Bz + PHLPPi and 20 nM Bz + PHLPPi, which reduced the viability of cells by 31% (*p* = 0.0001) and 44% (*p* = 0.0001), respectively.

In summary, the inhibition of CMA sensitises resistant cells to Bz, and the inhibition of CMA enhanced the cytotoxic effects of Bz when used in combination to treat both Bz-sensitive and resistant cells. Therefore, CMA may confer Bz resistance in MM and inhibition of CMA may sensitise resistant cells to Bz.

## 4. Discussion

With the introduction of Bz, the life expectancy of MM patients has improved significantly. However, while Bz has been an effective drug for treating the disease, MM still remains an incurable malignancy. Even though the majority of patients eventually develop resistance, a significant proportion of MM patients have de novo resistance to Bz [27]. The causes of Bz resistance in MM still remains largely unknown, although it is likely that an alternative stress mechanism is responsible in conferring resistance. Within this study, we have presented in vitro and ex vivo evidence that upregulation of CMA is an important mechanism in conferring Bz resistance, and the inhibition of CMA in vitro resensitises resistant cells to Bz.

Through MM Bz-resistant cell line modelling and the analysis of ex vivo patient samples, we demonstrated the importance of CMA in Bz resistance in MM as a mechanism conferring Bz resistance. The protein expression of LAMP2A, the rate limiting factor of CMA activity, was significantly increased in patients who were resistant to Bz. Resistant patients also displayed an increasing trend in LAMP2A mRNA expression compared to the sensitive patient cohort. However, due to the small sample size and variance of the mRNA levels between the individual patients, the increase in expression was not statistically significant. Furthermore, increases in LAMP2A expression also coincided with CMA activity within the resistant cell line model. Resistant cells possessed substantially higher CMA levels compared to the parent sensitive cells under basal conditions, almost reaching maximum active capacity. These findings and LAMP2A expressional results converge to suggest the importance of CMA in Bz-resistant MM and a possible driving factor in conferring Bz resistance. Breast cancer cells deficient of LAMP2A have previously been found to have increased sensitivity to the drug doxorubicin [11], suggesting the importance that CMA plays in conferring chemotherapy resistance. Therefore, our findings are the first to confirm the importance of CMA in Bz resistance in MM.

Furthermore, elevated CMA levels could also be attributing to many other aspects of the functioning and survival of MM cells. Alterations to CMA activity have been shown to change the glucose and lipid metabolism of cells [9,15,28,29,30]. In addition to metabolic regulation, it is plausible that CMA could be indirectly promoting NF-κB (nuclear factor kappa-light-chain-enhancer of activated B cells) activity by degrading IκBα (nuclear factor of kappa light polypeptide gene enhancer in B cells inhibitor, alpha), a known substrate of CMA [31,32]. IκBα inhibits and regulates NF-κB, preventing DNA transcription, cytokine production and cell survival [33,34], and is often dysregulated within MM and many other cancers [35,36]. It is likely that CMA is further driving MM progression via increasing NF-kB activity. It is therefore highly conceivable that the importance of CMA is not confined to alleviating ER stress in Bz resistance, but also plays a role in MM biology associated with metabolic regulation, progression and cell survival. Therefore, it would be of great benefit in future research to identify the pathways associated with CMA by evaluating pathway alterations following CMA using mass spectrometry.

Through in vitro cell line modelling, we demonstrated the potential therapeutic benefit of targeting CMA in the treatment of Bz-resistant MM. In combination with Bz, the inhibition of CMA using a PHLPP1 inhibitor, a positive regulator of the pathway, further enhanced the cytotoxic effect of Bz in MM. Inhibition of CMA sensitised resistant cells to Bz and resulted in significant cytotoxicity in Bz-resistant cells compared to Bz alone. As illustrated in the literature, myeloma cells resistant to Bz have reduced dependence on the UPR [37,38], reflected by the substantial decreases in XBP-1 and ATF6 expression [5,6]. Reduced UPR activity and proteasome inhibition is most likely making resistant cells depend on CMA as an alternative stress mechanism for compensating the UPR and alleviating ER stress. The inhibition of CMA would therefore sensitise resistant cells to proteasome inhibition, resulting in lethal proteotoxicity, which is the likely explaination as to why the combination of Bz and PHLPPi resulted in a more rapid induction of apoptosis compared to Bz treatement alone. These findings have therefore provided evidence to suggest CMA as a novel mechanism in Bz resistance and as a promising therapeutic target for overcoming Bz resistance.

CMA is known to be upregulated in response to various stress conditions such as oxidative stress and nutrient starvation and is also involved in eliminating damaged proteins [13,14,15]. Our data has further indicated an additional role of CMA involved in modulating ER stress. Myeloma cells sensitive to Bz have been recognized as being primarily dependent on the UPR to maintain proteostasis and to reduce ER stress attributed to accumulating paraprotein. We identified that CMA is an important compensatory stress mechanism capable of alleviating ER stress and restoring cellular homeostasis in MM through the upregulation of the pathway during ER-induced stress. Upregulation occurred in response Bz and DTT, two ER stressors with different mechanisms of action, which were reflected by an increase in LAMP2A protein expression and an increase in CMA active lysosomes. This has therefore established a link between the UPR and CMA in myeloma. This novel role in managing ER stress establishes an additional role that CMA has, and potentially further explains how myeloma cells manage cellular stress attributed to Bz treatment. In future research, it would be of benefit to identify the mechanisms driving the upregulation of CMA through LAMP2A expression. In doing so, this could potentially identify new drugable targets and provide the foundation for the development of novel therapies that could be used to treat MM.

Overall, this study has improved the understanding of Bz resistance in MM, deciphered the role of CMA in MM and Bz resistance and more importantly has discovered a novel therapeutic target that could be potentially used in the treatment of MM. Further work exploring the therapeutic benefit of targeting CMA in MM is worthwhile and promising.

## Data Availability

The data presented in this study are available on request from the corresponding author. The data are not publicly available due to privacy issue.

## Supplementary Materials

The following are available online at https://www.mdpi.com/article/10.3390/cells10123464/s1, Table S1: Patient characteristics; Figure S1: Apoptotic cell populations following CMA inhibition in sensitive and resistant KMS11 cell lines.
