# Supplementary material for: The Role of Chaperone-Mediated Autophagy in Bortezomib Resistant Multiple Myeloma
Source: Cells. 2021 Dec 8;10(12):3464. doi: 10.3390/cells10123464 (PMC8700264; doi:10.3390/cells10123464)
Supplement: Supplementary file 1 [file cells-10-03464-s001.zip › cells-1304316-supplementary/cells-1304316-supplementary.pdf]

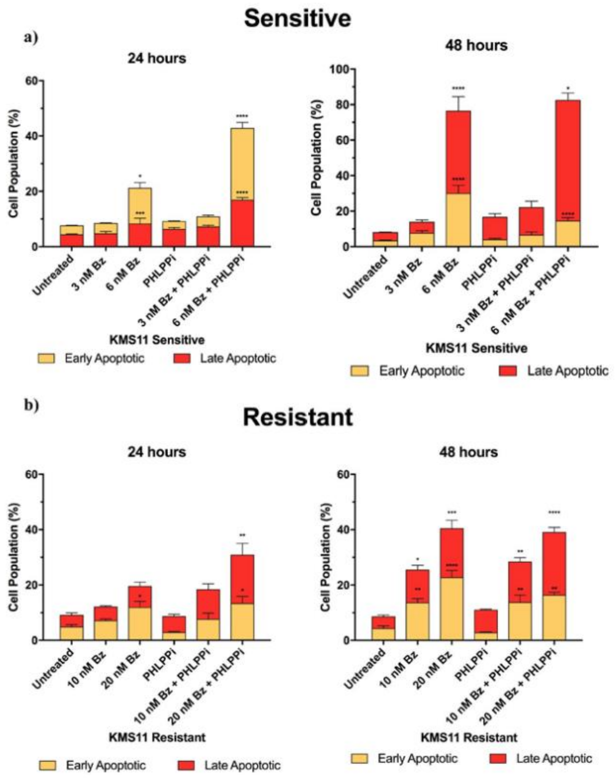

Supplementary Figure S1: Apoptotic cell populations following CMA inhibition in sensitive and resistant KMS11 cell lines. Apoptotic cells were analysed at 24 and 48 hour time points by flow cytometry using PE-Annexin V (apoptotic cell marker) and 7-AAD (live cell exclusion stain) staining. For each experiment, a total of 10,000 cells were analysed per sample. Early apoptotic (annexin V: positive/7AAD: negative) (yellow bar) and late apoptotic (annexin V: positive/7AAD: positive) (red bar) cell populations of bortezomib sensitive and resistant KMS11 cell lines treated with either bortezomib, PHLPPi, or in combination with PHLPPi and bortezomib. Data is presented as mean population percentage ± S.E.M (n=3). Statistical analysis was performed using a one-way ANOVA across all samples a) KMS11 sensitive cells (24h: Early apoptotic ***p=0.0004; ****p=≤0.0001; Late apoptotic *p=0.0484; ****p=≤0.0001) (48h: Early apoptotic ****p=≤0.0001;

*p=0.0112; Late apoptotic ****p=≤0.0001; ****p=≤0.0001). b) KMS11 resistant cells (24h: Early apoptotic *p=0.0387; *p=0.0129; Late apoptotic **p=0.002) (48h: Early apoptotic **p=0.0071; ****p=≤0.0001; **p=0.0065; **p=0.001; Late apoptotic *p=0.0244; ***p=0.0003; **p=0.003; ****p=≤0.0001).
