# Supplementary material for: The Role of Chaperone-Mediated Autophagy in Bortezomib Resistant Multiple Myeloma
Source: Cells. 2021 Dec 8;10(12):3464. doi: 10.3390/cells10123464 (PMC8700264; doi:10.3390/cells10123464)
Supplement: Supplementary file 1 [file cells-10-03464-s001.zip › cells-1304316-supplementary/Supplementary+Data+Table+1.pdf]

Supplementary data

Table S1. Patient characteristics

Patient characteristics	Total (n = 29)
Age, years (mean ± SD)	63 ± 10.15
Newly Diagnosed MM	18 (62.1%)
Relapsed MM	11 (37.9%)
Myeloma Subtypes	
IgG	16 (55.2%)
IgA	3 (10.3%)
IgM	0
Light Chain Disease	10 (34.5%)
Kappa	3 (10.3%)
Lambda	7 (24.1%)
International Staging System	
Stage I	12 (41.4%)
Stage II	5 (17.2%)
Stage III	5 (17.2%)
Unknown	7 (24.1%)
